# NEMix: Single-cell Nested Effects Models for Probabilistic Pathway Stimulation

**DOI:** 10.1371/journal.pcbi.1004078

**Published:** 2015-04-16

**Authors:** Juliane Siebourg-Polster, Daria Mudrak, Mario Emmenlauer, Pauli Rämö, Christoph Dehio, Urs Greber, Holger Fröhlich, Niko Beerenwinkel

**Affiliations:** 1 Department of Biosystems Science and Engineering, ETH Zurich, Basel, Switzerland; 2 SIB Swiss Institute of Bioinformatics, Basel, Switzerland; 3 Institute of Molecular Life Sciences, University of Zurich, Zurich, Switzerland; 4 Biozentrum, University of Basel, Basel, Switzerland; 5 Algorithmic Bioinformatics, Bonn-Aachen International Center for IT, University of Bonn, Bonn, Germany; University of Tokyo, Japan

## Abstract

Nested effects models have been used successfully for learning subcellular networks from high-dimensional perturbation effects that result from RNA interference (RNAi) experiments. Here, we further develop the basic nested effects model using high-content single-cell imaging data from RNAi screens of cultured cells infected with human rhinovirus. RNAi screens with single-cell readouts are becoming increasingly common, and they often reveal high cell-to-cell variation. As a consequence of this cellular heterogeneity, knock-downs result in variable effects among cells and lead to weak average phenotypes on the cell population level. To address this confounding factor in network inference, we explicitly model the stimulation status of a signaling pathway in individual cells. We extend the framework of nested effects models to probabilistic combinatorial knock-downs and propose NEMix, a nested effects mixture model that accounts for unobserved pathway activation. We analyzed the identifiability of NEMix and developed a parameter inference scheme based on the Expectation Maximization algorithm. In an extensive simulation study, we show that NEMix improves learning of pathway structures over classical NEMs significantly in the presence of hidden pathway stimulation. We applied our model to single-cell imaging data from RNAi screens monitoring human rhinovirus infection, where limited infection efficiency of the assay results in uncertain pathway stimulation. Using a subset of genes with known interactions, we show that the inferred NEMix network has high accuracy and outperforms the classical nested effects model without hidden pathway activity. NEMix is implemented as part of the R/Bioconductor package ‘nem’ and available at www.cbg.ethz.ch/software/NEMix.

## Author Summary

Experiments monitoring individual cells show that cells can behave differently even under same experimental conditions. Summarizing measurements over a population of cells can lead to weak and widely deviating signals, and subsequently applied modeling approaches, like network inference, will suffer from this information loss. Nested effects models, a method tailored to reconstruct signaling networks from high-dimensional read-outs of gene silencing experiments, have so far been only applied on the cell population level. These models assume the pathway under consideration to be activated in all cells. The signal flow is only disrupted, when genes are silenced. However, if this assumption is not met, inference results can be incorrect, because observed effects are interpreted wrongly. We extended nested effects models, to use the power of single-cell resolution data sets. We introduce a new unobserved factor, which describes the pathway activity of single cells. The pathway activity is learned for each cell during network inference. We apply our model to gene silencing screens, investigating human rhino virus infection of single cells from microscopy imaging features. Comparing the learned network to the known KEGG pathway of the genes shows that our method recovers networks significantly better than classical nested effects models without capturing of hidden signaling.

*This is a PLOS Computational Biology Methods paper.*

## Introduction

Network inference benefits substantially from perturbation experiments, such as RNA interference (RNAi) screens. Monitoring high-dimensional effects of gene silencing enables inference of non-transcriptional network structures that cannot be learned on observational data alone [1]. Nested effects models (NEMs) are a class of probabilistic graphical models that aim at learning hierarchical dependencies from such intervention experiments. Upon perturbing nodes in a signaling graph, their connectivity is inferred from the nested structure of observed downstream effects. The concept was first introduced in [2]. Since then, many further additions concerning, for example, parameter inference, structure learning, and data integration, were developed [3, 4]. In addition, dynamic models for time series data have been developed [5–7]. In [5], a first application of dynamic nested effects models to time laps microscopy data has been described, but the model can not handle single-cell data. A Bayesian network representation of NEMs in [8] introduces a probabilistic notation for signal propagation, but in practice the signaling is kept deterministic. In all previous NEM models and applications, the signaling pathway under observation is assumed to be active and the signal flow disrupted by silencing the signaling genes one by one.

In principle, RNAi experiments are a highly informative for learning NEMs. Perturbations are introduced by gene silencing in cells through RNA interference using siRNAs [9, 10]. Effects of the knock-downs are then captured by high-dimensional down-stream observations. The screening data analyzed here, comprises imaging data of thousands of individual cells for genome-wide gene silencing. However, the experiments come at the cost of high noise levels, as well as biological and technical biases, including off-target effects [11, 12]. These confounding factors complicate the analysis and interpretation of the screening results. On the other hand, RNAi screens currently reach very high resolution. Per knock-down, the present data sets comprise about 300 image features for several hundred individual cells, which allows for a very detailed analysis of a knock-down event. However, it has been shown that measurements from individual cells of the same experiment can differ widely, for example, due to local environmental differences [13, 14]. Such variation on the single cell level needs to be accounted for. Otherwise, an ambiguous signal is obtained, when averaging over the cell population of a knock-down.

Here, we specifically investigate single-cell observations of pathogen infection screens [15–17]. The experiments monitor cells with an siRNA knock-down during infection with human rhinovirus (HRV). After siRNA knock-down, the pathogen is added to the cells, and the success of infection as well as many other cellular features are extracted from microscopy images taken of the cells from each experiment [18–20]. The aim is to infer a signaling cascade involved in pathogen entry in to the host cell. However, a challenge in the analysis of data from this experimental setup is that by experimental design even in mock controls (i.e., infection without knock-down) the infection rate is far from complete. In fact, the multiplicity of infection (MOI) of the assay was optimized to reach 30 to 50% infected cells, such that both infection-decreasing and infection-increasing hits can be detected. Which cells in the population finally get infected is, at least to some extent, the result of stochastic effects, since cellular processes can be differently manifested in different cells. The multi-functional nature of proteins, for instance, enables a single host factor to enhance a signaling cascade, and at the same time may antagonize other processes that support or inhibit infection. Obviously, infected cells were reached by a pathogen triggering some signal to get internalized. However, for uninfected cells, it is unknown whether a pathogen actually attempted to infect them, which is crucial for determining the effect that the gene knock-down had on these cells. Wrongly assuming that the pathway is active, even though it is not, can result in conflicting knock-down schemes. In the original NEM setting, individual cell observations are summarized for each signaling gene.

To address the problem of network learning when the activation state of the signaling pathway is unknown we introduce a new model, called NEMix, extending the existing NEM framework in several ways. First, we do not summarize the data across cells, but rather perform network inference using the single-cell observations directly. Furthermore, we model the unknown pathway activation with an additional hidden random variable in the graph of signaling genes. The activation state is then estimated for each individual cell. The pathway activity can be regarded as an additional hidden silencing event in the signaling graph. We introduce a general theoretical framework for probabilistic combinatorial knock-downs in NEMs. We develop our model for the most general case, not making any assumptions about the signal propagation. We have implemented the special case of one hidden variable with probabilistic knock-down, where the remaining network is kept deterministic. For inference of the hidden pathway state, we developed an EM algorithm [21]. This step is repeated for each proposal structure during the network search.

## Results

### Network inference under unknown pathway activity

**Fig 1:**
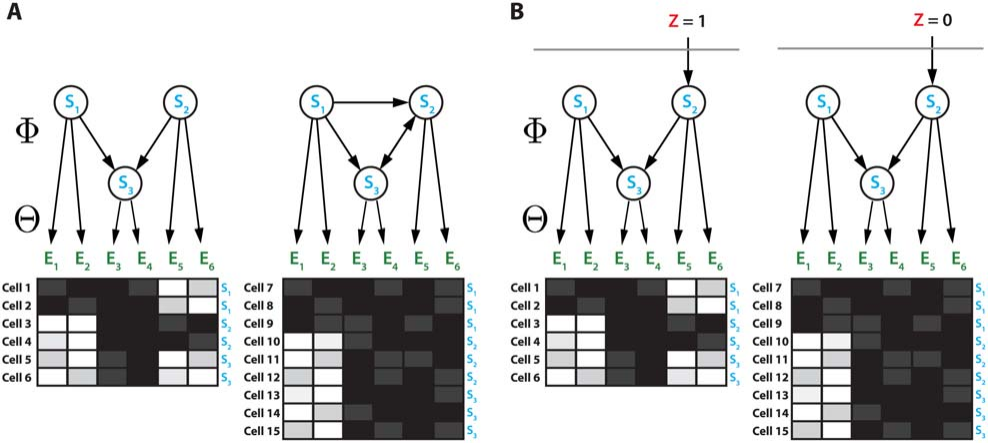
NEM versus NEMix. A schematic example is shown comparing the classical nested effects model (NEM; panel **A**) with the new nested effects mixture model (NEMix; panel **B**) on six features observed in 15 individual cells. Blue nodes in the graph depict the signaling genes *S*_1_, *S*_2_, and *S*_3_ that have been silenced and whose dependency structure is sought. The observed features *E*_1_, …, *E*_6_ are shown in green. Each box below the graphs indicates the observed (noisy) features (e.g., image-based read-outs) for a single cell. Within each box, dark entries indicate an effect of the knock-down on the feature, light entries indicate no effect. In cells 1 and 2 (left in both **A** and **B**), the pathway has been activated via *S*_2_, whereas in cells 3, 4, and 5 (right in both **A** and **B**) it has remained inactivated. In the latter case, the effects of silencing *S*_2_ are masked and the resulting silencing scheme then differs from the one where the pathway is stimulated. Classic NEMs (**A**) could explain such a heterogeneous cell population only by two different signaling graphs Φ. By contrast, with the NEMix model proposed in this work (**B**), both observed patterns can be explained by the same signaling graph Φ, because the hidden pathway stimulation *Z* (shown in red) is modeled explicitly. In the NEMix model, *Z* is a hidden binary random variable indicating pathway activation (*Z* = 1), which occurs with probability *P*(*Z* = 1) = *p*_1_.

We developed NEMix, a new model based on NEMs, which allows to estimate activity of a pathway in individual cells. A NEM is a graphical model, consisting of two graphs. The transitively closed graph Φ encodes dependencies among signaling gene nodes *S*_*s*_ ∊ **𝓢**, which are silenced one by one. The bipartite graph Θ connects a set of observable feature nodes *E*_*e*_ ∊ 𝓔 uniquely to the signaling genes (Fig. 1A). We seek the structure of Φ, i.e., the topology of the signaling pathway, by inferring it from the nested structure of observed effects. For a data set **𝒟** = (*d*_*ek*_) of a set of knock-down experiments *k* ∊ {1, …, *K*} and observed features *e* ∊ {1, …, *m*}, the likelihood function given Φ and *θ* is

$$\begin{array}{c} P\left(𝒟|\Phi ,\theta \right)=\prod_{e=1}^{m}\prod_{k=1}^{K}P\left(d_{ek}|\Phi ,\theta_{e}=s\right), \end{array}$$ (1)

where *θ*_*e*_ = *s* indicates that feature *e* is connected to signaling gene *s* ∊ **𝓢**.

The NEMix model consists of the same two graphs Φ and Θ, but has an additional binary hidden variable *Z* added to the signaling graph Φ. Its connections to the signaling genes, as well as its overall knock-down probability *p*_0_ = *P*(*Z*_*kc*_ = 0), are unknown and inferred for each individual cell during the network reconstruction process. Given single cell data **𝒟** = (*d*_*ekc*_) with *c* = 1, …, *c*_*k*_ cells in knock-down experiment *k*, the likelihood function of the NEMix model, given Φ and *θ*, is

$$\begin{array}{c} P\left(D|\Phi ,\theta \right)=\prod_{e=1}^{m}\prod_{k=1}^{K}\prod_{c=1}^{c_{k}}\sum_{j∊\{0,1\}}p_{j}P\left(d_{ekc}|\Phi ,\theta_{e}=s,Z_{kc}=j\right). \end{array}$$ (2)

A detailed derivation of the model and its implementation are given in the Models section. If a signal is activating a pathway, or parts of it, the signal flow is the same as in the NEM. Also the observed knock-down effects for the features *E*_*e*_ are the same. However, when the pathways input signal is inactivated, the knock-down pattern of the features changes (Fig. 1A and B, cells 7 to 15). Not accounting for the pathway disruption can mislead inference of the structure Φ (Fig. 1A, left model).

The connectivity of *Z* is learned in a greedy fashion during structure inference. For the knock-down probability of the hidden variable, *p*_0_, we implemented an EM algorithm, which estimates jointly *p*_0_ from each cell’s observation and the connections of observations to signaling genes, *θ*. In the following, we show improved network inference with NEMix in simulations and then infer networks of high accuracy, from single cell gene silencing experiments.

### Simulation study

To test our model, we performed a large simulation study. We generated 30 network structures with 5 signaling genes, randomly sampled from KEGG pathway maps [22] as previously described in [6]. To each network the hidden input signal was attached randomly. The resulting 30 sample networks are shown in supplementary S1 Fig. From each network, we sampled 50 data sets on 300 observed features in the following way. For each gene, we simulated single knock-downs in 200 cells. To the observed features we added another 30 noise features, not attached to any signaling gene. The data sets were generated in the following way. We sampled effects from a normal distribution with mean *m*_*e*_ = 1 and non-effects from a normal distribution with mean *m*_*n*_ = 0. The standard deviation for each experiment was sampled uniformly between 2 and 2.5. We furthermore sampled 200 cells for control experiments. The negative control cells do not show any effects and are therefore drawn from the non-effect distribution. The positive control cells always show effects and hence are drawn from the effect distribution. The whole simulation process was repeated for five different fractions of pathway disruption, *p*_0_ ∊ {0, 0.3, 0.5, 0.8, 1}. NEMix inference was restarted for 16 initial networks. Each of them consists of the empty graph Φ plus a unique attachment of *Z* to the signaling genes. Setting the maximal out-degree of *Z* to two, there are 16 possible such attachments of *Z*. This regularization on the edges of *Z* reduces the search space significantly. During structure search we also imposed this restriction, but additionally allowed transitive edges that had to be added as a consequence of the insertion of any edge connecting *Z* to a gene (see Models section).

We compared NEMix to two other NEM models and, for a baseline comparison, to a random approach, where network edges are sampled uniformly with probability 1/*n*, where *n*∣Φ∣ is the number of signaling nodes. This probability was chosen as it creates networks with approximately the same number of edges as in the original graphs. To assess the impact that pathway disruption has on the cell population level, we ran the simulations on a standard NEM using the log-likelihood model introduced in [23]. For the NEM approach we had to summarize the single cell observations to the gene level. For these gene-level data sets we used p-values of a Wilcoxon test comparing the cell population of a knock-down to the control distribution. From the p-value distributions a Beta-Uniform-Mixture model was estimated. For each feature a density value is calculated from this model, indicating the effect strength of the knock-down. These density values are used as the input data, as previously introduced in [23]. The third approach, called single-cell NEM (sc-NEM). is a NEMix model on individual cell observations, but with fixed *p*_0_ = 0, i.e., a single-cell observation-based NEM without considering uncertain pathway activity. For all three models, we applied a uniform prior on the feature attachments *θ*, and no prior knowledge was added for the network structures Φ. The NEMix parameter *p*_0_ was initialized by drawing from a uniform distribution in each EM restart. As NEMix and sc-NEMs infer networks on single-cell observations, we calculated log odds ratios from each observation based on the positive and negative control distributions (see ‘Modeling the effect likelihoods’ in S1 Text). For NEMs and sc-NEMs, we used maximum likelihood estimation to infer *θ* and in the NEMix it is estimated by in an EM algorithm. Structure learning is performed using a greedy hill climbing algorithm, initialized with an empty network.

**Fig 2:**
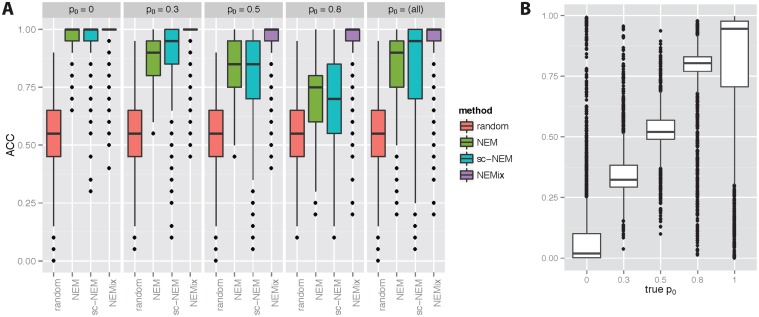
Performance comparison of the simulations. (A) Simulation results are summarized based on the accuracy of recovered edges for the compared methods. The methods are random, random edge sampling with rate $\frac{1}{n}$; NEM, the normal NEM inference; sc-NEM, the cell level NEM and NEMix, using the NEMix inference with the hidden pathway state. All methods were run on 50 simulated data sets from 30 sample networks, repeated for different knock-down probabilities of the pathway state *p*_0_. (B) For the NEMix model, the distributions of inferred *p*_0_ values are compared to the true *p*_0_.

Fig. 2A summarizes the overall performance for all methods and the different fractions of pathway signal perturbation *p*_0_. We display accuracy of the edge recovery, for varying *p*_0_. We also calculated the area under the ROC curve (AUC) based on the edge frequencies of the 50 replicate data sets, which yielded similar results in terms of accuracy (see supplementary S2 Fig). As expected, all methods performed equally well when there is no signal disruption (*p*_0_ = 0). However, when *p*_0_ is moderate to high, NEMix performs significantly better than the other methods. If the triggering signal is always turned off, performance of all methods drops drastically. Intuitively, this is because in such a special case, all features downstream of *Z* always show an effect and hence they cannot be used for structure learning. For example, if, in Fig. 1B, *Z* is inactive for each cell, we could not infer the structure among *S*_2_ and *S*_3_. In reality though, permanent shut down of the pathway is very unlikely. For the infection screens *p*_0_ = 1 would mean that no cell is ever infected. Pathway activity estimates are also of overall high accuracy (Fig. 2B). Although simulation results demonstrate that the performance of learning *Z* and *θ* varies, depending on the network structure, the average performance is very good (S3 Fig, S4 Fig, S5 Fig).

Currently, one of the main obstacles for learning larger NEMix models is the fast growing run-time for *n* > 5 network nodes. Run-time is further increased by a factor of *n*, when initiating the algorithm with each possible connection of *Z* to one of the knock-down genes. To assess its performance on larger networks, we ran a reduced simulation study on *n* = 5, 10, and 15 genes. The setup and results of the study are described in detail in S6 Fig. Larger networks of 15 nodes can still be estimated very well (S6 Fig. A) and estimation of the parameter *p*_0_ even improves (S1 Fig. D). However, the average time to estimate a 15-node network was 9.5 hours. This is substantially more than the average 1.9 hours needed for 10-node networks. Thus, in a highly parallelized computing environment, even larger networks can be estimated.

We also assessed the connection of features to the signaling genes in the inferred graph Θ. There can be situations, where attachment of features is equally likely for several signaling genes. In these cases, where no single gene is preferred, we counted a feature as correctly attached if it was connected to any of the signaling genes with equal likelihood. Accuracy of the *θ* estimates is high (> 80%) for small *p*_0_ values and decreases with increasing *p*_0_. For small *p*_0_, also performance of the sc-NEMs is good, which shows the advantage of learning on the single-cell data level. However, NEMix stands out from the other methods for higher *p*_0_. Recovery of noise features, i.e., correct filtering of the additionally added uninformative features, is not strongly affected by the hidden signal (see supplementary S7 Fig). Analyzing individual networks, one again observes high variation in performance (see supplementary S8 Fig).

### Application to pathogen infection experiments

We applied NEMix in the context of infection signaling, using the RNAi screening data monitoring HRV infection, mentioned in the introduction. Briefly, viruses were added to the siRNA transfected cells and after an incubation time, cells were fixated, stained, and then imaged. Subsequently, 360 cell features were extracted from the 9 images per knock-down experiment using the software CellProfiler [24]. For the whole experimental procedure the protocols of [17] were followed. The HRV assay is rather short with an infection time of only seven hours, resulting in measurements proximal to the infection event. The short time range is advantageous, because it leaves less room for confounding developments in the cells. Furthermore, the used antibody resulted in clean readouts, well to extract from the images.

Before using the data for network inference, we performed two additional filtering steps. For each knock-down, the well is split into 9 images. They are arranged in three rows and three columns. We used only the middle image, because it is of the highest quality. In this way we avoided too many out-of-focus cells, which bias especially the cell texture features. After this filtering step, we had around 200 to 300 cells per knock-down. A second filtering step concerns siRNA off-targets [25]. We sought to avoid confounding by this effect and therefore selected only genes with low predicted off-target effects as described in ‘siRNA filtering for off-targets’ of S1 Text.

We applied NEMix to a small subset of the screened genes, in order to recover a known pathway. We decided on the well-known MAP-Kinase signaling cascade as a proof of principle, for several reasons. First, it has been studied and validated in great detail [26–28], such that the available signaling network from the KEGG database [22] can be used as a reliable source to compare to. Second, the pathway is known to be involved in HVR infection signaling, where it is associated with asthmatic and COPD exacerbation [29–31]. Finally, we observed an enrichment for low off-target siRNAs in this pathway when performing a gene set enrichment analysis [32] (see supplementary S9 Fig). We then selected a small subset of 8 MAP-Kinase pathway genes for analysis based on the derived score for predicted off-target effects. Nodes of KEGG pathways can contain several genes. We selected genes such that they are all assigned to different KEGG nodes using a weighted maximum bipartite matching of low off-target siRNAs and unique KEGG nodes. After gene selection, we inferred networks for the 5 and 8 genes with lowest off-target score.

Like in the simulation study above, we compared the NEMix model to the NEM and the sc-NEM approach. As input data sets, the local effect likelihoods from the selected knock-down gene experiments were computed as follows. As the experiments lack reliable controls, we instead used a random sample of cells from the plate on which the gene was located, assuming that the majority of knock-downs will not have an effect. Like for the simulation study, we derived the cell population effects for the NEM from Wilcoxon tests, comparing the knock-down experiment to the control. From the resulting p-value distributions, effect strengths for the features were estimated using the Beta-Uniform-Mixture model. Log odds ratios for sc-NEMs and NEMix in this case are calculated only based on one control distribution (see Models section). NEMix inference again is repeated for the 16 initial networks of all possible connections of *Z* with maximal out-degree 2 to the empty graph Φ. Like in the simulation study, *p*_0_ was initialized by drawing randomly from a uniform distribution. Again we used uniform priors for *θ* and imposed no priors for the signaling networks other than the maximal out-degree of *Z* (plus the transitive edges that need to be added).

**Fig 3:**
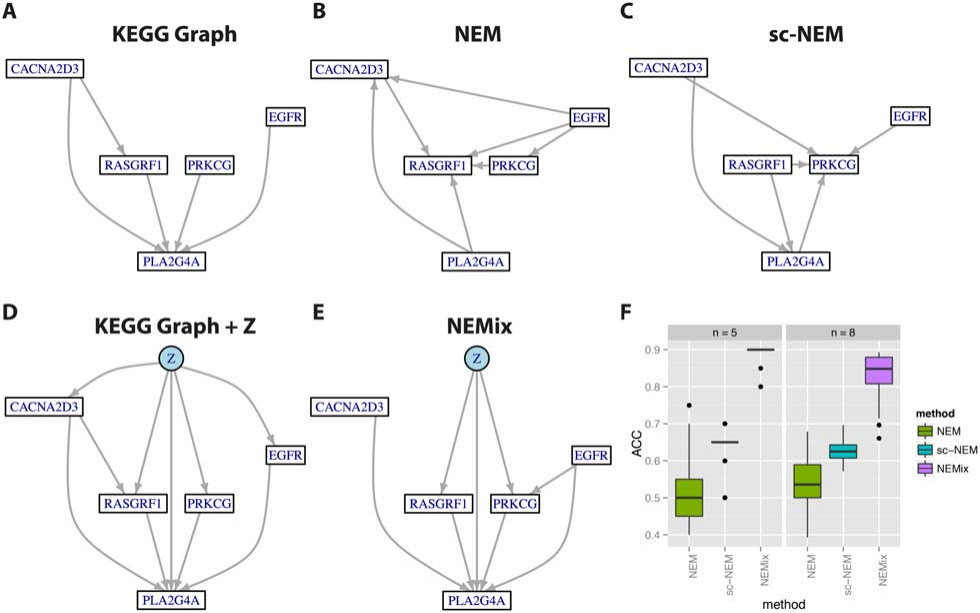
Inferred MAPK networks on HRV infection data. Best networks of the 5 top scoring siRNAs from the MAPK pathway for HRV infection for the different compared methods are displayed. (A) shows the known KEGG pathway. (B) is the inferred NEM and (C) the sc-NEM. (D) left shows the known network with the most likely attachment of the hidden variable *Z* (blue) and (E) is the inferred NEMix. For all networks their performance is summarized in Table 1. Subfigure (F) summarizes robustness of the MAPK network inference. For the inferred MAPK signaling networks on the HRV infection data, we assessed robustness of the accuracy for edge recovery. Box-plots display the result of 50 bootstrap samples for the three compared methods, on the 5 gene (*n* = 5) and 8 gene (*n* = 8) network.

The known KEGG network and the inferred results for the top 5 signaling genes are displayed in Fig. 3A-E. Results for the top-8 gene network are given in S10 Fig. To assess robustness of the learned networks, we repeated the inference on 50 bootstrap samples of the original data set. Both networks show high AUC values and even better accuracy (see Table 1). As can be seen from Fig. 3F, network inference was very robust for the top-5 gene network. For the top-8 gene network, performance had a slightly higher variation. Individual plots for sensitivity and specificity are given in supplementary S11 Fig. A, B. Also the estimate of *p*_0_ shows only little variation (S11 Fig. C). In all cases, the likelihood score of the known KEGG network is much lower than for the best inferred networks, indicating that under the assumptions of our model, the data and the KEGG database do not perfectly agree. Possible reasons for this observation include our model missing to explain part of the data correctly, the KEGG database being incomplete, and inaccuracies in the data generating process. Nevertheless, the accuracy value of 0.85 for the learned NEMix outperforms all other methods. All edges contained in the learned NEMix models are of high robustness (> 80% for 5 genes, and > 70% for 8 genes). Consensus networks of the bootstrap results are shown in supplementary S12 Fig.

**Table 1:** Performance summary of the 5 gene MAPK network.

Network	Likelihood	ACC	AUC	p0	Sub-figure
True Graph	2641.47	1	1		A
NEM	2809.83	0.5	0.23		B
sc-NEM	29410.81	0.65	0.47		C
True Graph + Z	31768.94	1	1	0.48	D
NEMix	34982.87	0.9	0.84	0.42	E

The first column gives the log-likelihood for each model, showing that the true network is much less likely than the inferred networks. The second and third column show performance of the networks in terms of accuracy (ACC) and area under curve (AUC). The inferred *p*_0_ for the NEMix models is displayed in column four. Column five indicates the corresponding sub-figure of Fig. 3. The network ‘KEGG Graph + Z’ denotes the structure of the known KEGG network, where only the position of *Z, p*_0_, and *θ* are inferred.

Furthermore, the hidden root *Z* is attached to the same nodes in both the known KEGG graph and the estimated network for 5 genes. Also the inferred 8 node network connects *Z* to the same three genes. As genes were selected based on small off-target effects of their targeting siRNAs, they are not necessarily hits for HRV infection. However, of the selected genes EGFR [33], TAB2 [34] and CACNA2D3 [35] have been shown to be involved in this process.

All models have a built-in filter for uninformative features, which has been previously introduced in [36]. A comparison shows that averaged over the bootstrap samples, for all three methods, the set of used features largely agrees (supplementary S13 Fig and S14 Fig). The maximum likelihood attachments of features to the knock-down genes and the null node are shown in supplementary S15 Fig and S16 Fig, together with a detailed description of the different feature types. The inferred signaling disruption of *p*_0_ = 0.42 seems rather high. We compared this to the average infection rate in mock experiments, i.e., cells without siRNA knock-down. These resemble cases, where *Z* can be perturbed but none of the other signaling genes in the network. Mock wells from plates of the 8 genes used here, actually have a much higher percentage of uninfected cells, roughly in the range of 75 to 81%. However, this comparison should be taken with caution since control wells of these screens might have suffered from strong plate location bias, as they were located on the margins of the plate.

As a general observation, NEMix-inferred networks were sparser than those obtained from NEMs, because spurious edges introduced in the latter are correctly explained by hidden pathway activity *Z* in NEMix. Therefore, NEMix networks have increased specificity, which might come at the cost of some missing true edges. Especially the 8-gene networks inferred by NEM and sc-NEM are much denser than the known KEGG network. A sparse network is beneficial in the sense that it allows to focus on a small set of highly specific edges. For validation experiments, it is desirable to have a low false positive rate in the predicted interactions as usually only very few of these dependencies can be experimentally tested.

## Discussion

RNAi screens are known to be prone to many sources of noise and bias such that their analysis is highly challenging. Here, we have identified one confounding factor, namely heterogeneous signaling pathway activation within a cell population, and incorporated it directly into a novel probabilistic model for pathway reconstruction. To address the problem of unknown activation of signaling pathways during network inference, we have introduced a general framework, building on NEMs, to handle hidden combinatorial knock-downs in a probabilistic manner. With NEMix we provide an implementation for inference under unknown pathway stimulation. For the first time, image features are explicitly used on the single-cell level for NEM inference, acknowledging large cell-to-cell variation. We have demonstrated the advantages of NEMix over current NEMs in simulations and inferred highly accurate networks in a case study on HRV infection. Especially, when the underlying true signaling networks are expected to be sparse, NEMix is beneficial. It removes spurious edges introduced due to confounding factors and therefore reduces the false positive rate, a desired property when it comes to validation of edges.

A limitation of the current model formulation is the assumption of independent single cell observations. In reality, this assumption might not be met as cells can be biased due to their location and neighbors. Removing this bias either by normalization or explicit modeling, as for example in [14], could further improve the model. Furthermore, in the current data sets cells can be in different cell cycle states. Grouping them according their states may remove further biases, but this clustering task is itself very challenging.

Another general limitation of NEMs and NEMix models is that they cannot learn certain pathway features. From static data, NEMs cannot resolve any loop structures by construction. This is a general problem for network inference without time resolved data. Therefore, only performance statements based on comparing transitively closed pathways can be made. The sampled graphs in the simulation are already transitively closed and since the transitive closure is a feature inherent to all the models we compare, it should not influence the ranking based on their performance. Before comparing a network to the corresponding KEGG pathway, we also built its transitive closure. This fact should be considered when interpreting the inferred models. For example, the model does not allow for distinguishing a feed forward loop from a sequential cascade; however, the hierarchical order of genes in the network would remain the same, and this piece of information does already provide considerable insight into the biological processes. The way we have assessed performance here puts particular emphasis on this hierarchical structure of the network nodes.

Further improvements could be achieved during data preparation. Image segmentation is not always perfect and might introduce technical biases into data sets, adding more confounding factors. If data is not curated carefully, we risk to capture technical biases with the additional hidden variable in NEMix models. Another interesting aspect of the data sets deserving a more thorough analysis, is the nature of the image features themselves. Here, readouts have been used to infer the graph of signaling genes. However, one could investigate in more detail how features are grouped when attaching them to the signaling genes. Some features might not contribute useful information and could be filtered in advance, others might be redundant. Future projects could use the output of NEMix models and seek for biological interpretation of feature correlations.

In case of cell infection screens, infection efficacy was an obvious factor that needed to be addressed. However, the same idea could be applied to other sources of noise. For example, transfection efficacy of the knock-downs could be considered. Quality and efficacy of a knock-down can be quantified by mRNA levels (qPCR) or protein level (western blot analysis) of a gene. However, for high-throughput assays, such confirmation is not available for most gene knock-downs. In order to account for different siRNA transfection efficacies further hidden variables could be introduced. In contrast to the global *Z* variable introduced here, hidden knock-down rates would then be estimated for each gene individually. As a consequence, the complexity of the problem would increase substantially. Instead of one parameter, *n* (number of genes) parameters would have to be estimated. Furthermore, knock-down probabilities could only be estimated from a fraction of the observations (e.g., cells under the specific knock-down). Another drawback is that the increased number of hidden variables gives rise to identifiability problems when estimating infection efficacy in combination with the knock-down rates. For example, if the hidden variable *Z* was only attached to one signaling gene, effects of *Z* and a failed transfection could not be distinguished. Although extending the NEMix model to this situation would be an interesting future project, we believe that problems in the transfection process play an overall minor role. For the current experiments, KIF11 siRNAs (cell killers) were used to control transfection quality on the plate level. For the plates containing the cells used in our analysis, these controls show very high penetrance, i.e., out of an average of 2000 cells per well, on average only 7% of cells survive in these wells. Although this test does not make a statement about the efficacy of individual siRNAs, it ensures the general functioning of the transfection process. Additionally, the library vendor claims the knock-down efficacies achieved with their smart-pool siRNAs to be in the range of 70–95%. This proportion is a result of many possible sources of imperfect gene silencing, including non-transfected cells and off-target effects. Given the above facts in combination with our off-target filtering strategy, we are convinced that the analyzed data are of high quality.

We tried to minimize the general problem of confounding siRNA off-targets by considering only genes targeted by siRNAs with low predicted off-target effects. This selection step helps to achieve reasonably unbiased results with our model, but it also limits the gene sets we can analyze. Ideally, we want to be able to select any gene of interest. This scenario calls for models that can correct the off-target effects on the single-cell level. A potential solution to this issue could be delivered by NEMs directly. We could still learn the networks based on siRNA knock-downs directly, but handle the signal propagation differently. With NEMix it is already possible to use each siRNA as a combinatorial knock-down. In reality however, individual genes are knocked-down to different degrees by an siRNA. In a NEM, this would mean to split up the silencing signal of an siRNA into partial knock-downs of several genes. Then, signal propagation would have to be formulated in a fully probabilistic fashion and NEMs would have to be reformulated such that their nodes do not have binary states anymore. Further developing NEMix, by integrating the above mentioned shortcomings, will make the models more powerful for future network reconstruction tasks.

Especially in the light of single cell data sets, which show large heterogeneity among individual observations, our approach is beneficial. Such data sets are becoming more and more available, and they reveal that the high cell-to-cell variation has severe consequences when summarizing such heterogeneous observations. On the population level, the signal is potentially confounded as it is only contained in part of the observations. NEMix uses the full power of single-cell experiments, as it is applied on the single-cell level directly, avoiding any data averaging. Only at this data resolution, the heterogeneity within a cell population can be accounted for and it becomes possible to investigate potentially confounding factors, such as, for example, pathway activity. NEMix is the first NEM-based method with additional unknown components in the signaling graph Φ. It is capable of inferring these missing data and provides an estimate for the fraction of signal disruption. We find such ambiguous signaling in RNAi infection screens and we have demonstrated that NEMix can improve network inference substantially by accounting for the confounding factor.

## Models

### The NEM framework

A NEM, as introduced in [2], aims to infer the hidden dependency structure among a set of *n* binary signaling variables **𝓢** from the nested structure of *m* observed effect variables 𝓔 (features). It therefore consists of two directed graphs, one describing the dependencies among the signaling genes and one connecting the features to the genes.

The binary adjacency matrix of signaling genes is denoted Φ = (*ϕ*_*ks*_), with *ϕ*_*ks*_ = 1 if gene *k* propagates its effects to gene *s* and using the convention Φ_*k, k*_ = 1, for all *k*. The signaling graph Φ is thus always transitively closed. If a gene is silenced, the effect is propagated deterministically along the edges of Φ. The connection of features 𝓔 to the genes **𝓢** is given by parameters *θ*_*e*_, where *θ*_*e*_ = *s* indicates that feature *e* is linked to gene *s*. For a gene *k* and a feature *e*, a NEM predicts an effect of *k* on *e* if there is a gene *s* such that *ϕ*_*ks*_ = 1 (i.e., *k* and *s* are connected), and *θ*_*e*_ = *s* (i.e., *s* has an effect on *e*). The observed data are denoted *D* = (*d*_*ek*_), where each *d*_*ek*_ is the measurement of feature *e* under perturbation of *k* (Fig. 1A).

Given an external signal which affects one or more of the signaling genes, each of them will have a binary signaling state. The state value is 0 if the signaling is interrupted, i.e., does not reach the node, and 1 if the signal reaches the node, i.e., the natural state of a stimulated pathway.

For inferring the structure Φ among the signaling genes, we consider its posterior

$$\begin{array}{c} P\left(\Phi |D\right)=\frac{P(D|\Phi )P(\Phi )}{P(D)}, \end{array}$$ (3)

where the marginal likelihood *P*(*D*∣Φ) can be obtained by integrating out the connections of features to the genes,

$$\begin{array}{c} P\left(D|\Phi \right)=\int_{\theta }P\left(D|\Phi ,\theta \right)P\left(\theta |\Phi \right)d\theta , \end{array}$$ (4)

with prior distribution *P*(*θ*∣Φ). In the absence of further knowledge, the prior is usually set to the uniform distribution. Given the network structure and assuming conditional independence of the parameters *θ*_*e*_ and of the silencing experiments *k*, the marginal likelihood becomes

$$\begin{array}{c} P\left(D|\Phi \right)=\prod_{e=1}^{m}\sum_{s=1}^{n}\left[\prod_{k=1}^{K}P\left(d_{ek}|\Phi ,\theta_{e}=s\right)\right]P\left(\theta_{e}=s\right). \end{array}$$ (5)

The local effect likelihoods *P*(*d*_*ek*_∣Φ, *θ*) denote the probability of observing an effect in feature *e* under knock-down of gene *k*. They can usually be pre-computed from the data and different approaches have been proposed [2, 23, 36]. For the results presented below, log-odds ratios as introduced in [36] were used (see ‘Modeling the effect likelihoods’ in S1 Text for details).

### The NEMix model

We first define the NEMix model and then derive it in detail. A NEMix consists of a nested effects model with effects graph Θ and an extended signaling graph Φ. The signaling graph Φ describes the dependency structure among the signaling genes and has an additional binary hidden variable *Z* indicating pathway activity. *Z* is a root of Φ, i.e., it can be connected to any of its nodes and does not have any direct connections to features in *θ*. The silencing probability of *Z* is denoted by *p*_0_ and is a priory not known. For a set knock-down experiments *k* ∊ {1, …, *K*}, with single cell observations *c* ∊ {1, …, *c*_*k*_} of signaling gens *s* ∊ {1, …, *n*} and features *e* ∊ {1, …, *m*}, the marginal likelihood of a NEMix is

$$\begin{array}{c} P\left(D|\Phi \right)=\prod_{e=1}^{m}\sum_{s=1}^{n}P\left(\theta_{e}=s\right)\prod_{k=1}^{K}\prod_{c=1}^{c_{k}}\sum_{j∊\{0,1\}}p_{j}P\left(d_{ekc}|\Phi ,\theta_{e}=s,Z_{kc}=j\right), \end{array}$$ (6)

where *p*_*j*_ = *P*(*Z*_*k*_ = *j*).

#### Probabilistic combinatorial knock-downs

We first extend the model to cope with several silenced genes at the same time. To achieve this goal, we condition the perturbation state of each gene on the states of its parents. For each knock-down experiment *k* ∊ {1, …, *K*}, let **𝓢**_*k*_ ⊆ **𝓢** be the set of genes knocked down at the same time in experiment *k*. We assume for combinatorial silencing events that we observe an effect on feature *e* if it can be reached by either of the genes in **𝓢**_*k*_ through a path in Φ. Let furthermore *S*_*sk*_ be the hidden binary random variable for the silencing state of gene *s* under knock-down of **𝓢**_*k*_. Then *S*_*sk*_ = 0 if the gene is perturbed and *S*_*sk*_ = 1 if it is not. If *s* ∊ **𝓢**_*k*_, then *S*_*sk*_ is set to zero but otherwise its value depends on the states of the parents of *s, S*_*pa*(*s*)*k*_ ∊ {0, 1}^∣*pa*(*s*)∣^, through the conditional probability *P*(*S*_*sk*_∣*S*_*pa*(*s*)*k*_).

The local effect likelihoods are then given by the marginalization over *S*_*sk*_,

$$\begin{array}{c} P\left(d_{ek}|\Phi ,\theta_{e}=s\right)=\sum_{x∊\{0,1\}}P\left(d_{ek}|\theta_{e}=s,S_{sk}=x\right)P\left(S_{sk}=x\right), \end{array}$$ (7)

where *P*(*S*_*sk*_) is the probability of gene *s* being active in experiment *k*. If the state *S*_*sk*_ depends on the states of the parent nodes, one can deduce the marginal *P*(*S*_*sk*_) from the joint distribution *P*(*S*) = *P*(*S*_1*k*_, …, *S*_*nk*_) of the signaling graph Φ. The joint probability factorizes when conditioning on the parent nodes,

$$\begin{array}{c} P\left(S_{sk}\right)=\sum_{S_{1,k},\ldots ,S_{s-1,k},\;S_{s+1,k},\ldots ,S_{n,k}}P\left(S_{1k},\ldots ,S_{nk}\right) \end{array}$$ (8)

However, for all signaling genes in **𝓢**_*k*_, we know that their state is 0, independent of their parents. So in fact, we only need to sum over all genes **𝓢**_*a, k*_ = {*S*_1, *k*_, …, *S*_*s*−1, *k*_, *S*_*s*+1, *k*_, …, *S*_*n, k*_} that are not in the set of knock-down genes **𝓢**_*k*_, except for gene *s* itself:

$$\begin{array}{c} P\left(S_{sk}\right)=\sum_{{𝓢}_{a}}\prod_{i\notin {𝓢}_{k}}P\left(S_{ik}|S_{pa(i)k}\right)\underset{=1}{\underset{︸}{\prod_{i∊{𝓢}_{k}}P\left(S_{ik}=0\right)}}. \end{array}$$ (9)

If *s* ∊ **𝓢**_*k*_, then *P*(*S*_*sk*_ = 0) = 1. Substituting (7) and (9) into the marginal likelihood (5) leads to

$$\begin{array}{c} P\left(D|\Phi \right)=\prod_{e=1}^{m}\sum_{s=1}^{n}P\left(\theta_{e}=s\right)\prod_{k=1}^{K}\sum_{x∊\{0,1\}}P\left(d_{ek}|\theta_{e}=s,S_{sk}=x\right)\sum_{{𝓢}_{a,k}}\prod_{i\notin {𝓢}_{k}}P\left(S_{ik}|S_{pa(i)k}\right). \end{array}$$ (10)

The conditional local effect likelihoods *P*(*d*_*ek*_∣*θ*_*e*_ = *s, S*_*sk*_ = *x*) can usually be pre-computed (see ‘Modeling the effect likelihoods’ in S1 Text).

#### Deterministic combinatorial knock-downs

If we assume deterministic signaling and all knock-downs are known, then *P*(*S*_*sk*_) will either be 0 or 1. As mentioned above, we assume a gene to be perturbed if at least one of its parents is perturbed, and unperturbed if none of the parents are perturbed. For transitively closed NEMs this also means *S*_*sk*_ = 0 if and only if **𝓢**_*k*_∩*pa*(*s*) ≠ ∅, i.e., only if the parents of *s* contain one of the knocked-down genes, then *s* itself can be perturbed. Therefore, the conditional probabilities *P*(*S*_*sk*_∣*S*_*pa*(*s*)*k*_) are in this case

$$\begin{array}{cc} & P(S_{sk}=1|S_{pa(s)k})=\left\{\begin{array}{cc} 1 & \text{if}\;(S_{pa(s)k}=(1,1,\ldots ,1)\;\text{or}\;pa(s)=\emptyset )\;\text{and}\;s\notin {𝓢}_{k}\\ 0 & \text{otherwise} \end{array}\right.\\ & P(S_{sk}=0|S_{pa(s)k})=1-P(S_{sk}=1|S_{pa(s)k}). \end{array}$$ (11)

Since these probabilities are either 0 or 1, we use the following indicator function

$$\begin{array}{c} \delta_{sk}≔P\left(S_{sk}=1\right)=P\left(S_{sk}=1|S_{pa(s)k}\right). \end{array}$$ (12)

The last equation holds, because for

$$\begin{array}{cc} P(S_{sk}) & =\sum_{S_{pa(s)k}}P\left(S_{sk},S_{pa(s)k}\right)=\sum_{S_{pa(s)k}}P\left(S_{sk}|S_{pa(s)k}\right)P\left(S_{pa(s)k}\right) \end{array}$$ (13)

all terms *P*(*S*_*pa*(*s*)*k*_) except for one parent configuration are zero. The local effect likelihoods can then be written as

$$\begin{array}{cc} P(d_{ek}|\Phi ,\theta_{e}=s) & =\sum_{x∊\{0,1\}}P\left(d_{ek}|\theta_{e}=s,S_{sk}=x\right)\delta_{sk}\\ & =\left\{\begin{array}{cc} P(d_{ek}|\theta_{e}=s,S_{sk}=0) & \text{if}\;\delta_{sk}=1\\ P(d_{ek}|\theta_{e}=s,S_{sk}=1) & \text{if}\;\delta_{sk}=1, \end{array}\right. \end{array}$$ (14)

which resembles the situation introduced in [6] for dynamic NEMs. However, in the following we make use of the more general case.

#### Hidden pathway stimulation

We now turn to a special case, where exactly one (root) node of the network has probabilistic signaling and the others follow the deterministic rules above. Silencing experiments can be noisy for many different reasons and it might be unknown whether the signaling pathway of interest is actually activated during knock-down of a gene. To model this uncertainty, we consider an additional hidden binary random variable *Z*_*k*_, indicating the state of an external signal that activates the pathway, where *Z*_*k*_ = 1 means active and *Z*_*k*_ = 0 means inactive in experiment *k*. The random variable *Z* can be viewed as an additional node in Φ that has only outgoing edges and can not have any observables directly attached to it (see Fig. 1B for an example). Let furthermore *p*_0_ = *P*(*Z*_*k*_ = 0) be the probability that the signaling pathway has not been activated, and *p*_1_ = *P*(*Z*_*k*_ = 1) = 1 − *p*_0_ the probability that it is active. The silencing of genes **𝓢**_*k*_ together with a unknown pathway stimulation can then be regarded as a hidden combinatorial knock-down event, where signaling genes **𝓢**_*k*_ are silenced deterministically and the external signal *Z*_*k*_ is inactivated with probability *p*_0_. Fig. 1B illustrates a simple NEMix with the additional pathway state variable. Since *Z* has no parents, we can easily factorize *p*_*j*_ out of the joint probability of the states *P*(*S*) in (9) to obtain

$$\begin{array}{c} P\left(S_{sk}\right)=\sum_{j∊\{0,1\}}p_{j}∙P\left(S_{sk}|Z_{k}=j\right)=\sum_{j∊\{0,1\}}p_{j}∙\delta_{sk}^{j}, \end{array}$$ (15)

where $\delta_{sk}^{j}=P(S_{sk}|Z_{k}=j)$ is again an indicator function for the state of *s* in experiment *k*, given that the pathway is in state *j*. Substituting this expression into the local effect likelihoods (7) leads to

$$\begin{array}{cc} P(d_{ek}|\Phi ,\theta_{e}=s) & =\sum_{S_{sk}∊\left\{0,1\right\}}P\left(d_{ek}|\Phi ,\theta_{e}=s,S_{sk}\right)\sum_{j∊\{0,1\}}p_{j}∙\delta_{sk}^{j}\\ & =\sum_{j∊\{0,1\}}p_{j}∙P\left(d_{ek}|\Phi ,\theta_{e}=s,Z_{k}=j\right), \end{array}$$ (16)

and the marginal likelihood (10) becomes

$$\begin{array}{c} P\left(D|\Phi \right)=\prod_{e=1}^{m}\sum_{s=1}^{n}P\left(\theta_{e}=s\right)\prod_{k=1}^{K}\sum_{j∊\{0,1\}}p_{j}P\left(d_{ek}|\Phi ,\theta_{e}=s,Z_{k}=j\right). \end{array}$$ (17)

For the RNAi infection experiments described in the introduction and the results, pathways of interest are those involved in infection signaling, i.e., pathways which are activated upon signals triggered by a pathogen. However, infection of a cell is a stochastic event, depending on many factors, for example, whether at all a pathogen docked on successfully to the cell. Consequently, in a cell with a silenced gene, there can be several explanations for why it stayed uninfected. It could be because the knocked-down gene was important for the infection signaling, but there is also a chance that other factors account for this, for example, no pathogen came within reach of the cell. In case a pathogen triggered a signal, the pathway is considered active (*Z*_*k*_ = 1), corresponding to a normal NEM. When no infection attempt was made, the infection pathway is inactive (*Z*_*k*_ = 0).

The population of cells in knock-down experiment *k* can be divided into infected and uninfected cells. For infected cells, the external input signal from the pathogen reached the cell and the signaling pathway is active (*Z*_*k*_ = 1). In these cases *Z* is observed. For uninfected cells, however the state of *Z*_*k*_ is unknown and no longer deterministic. So, for an infected cell, we have *P*(*Z*_*k*_ = 0) = 0 and *P*(*Z*_*k*_ = 1) = 1, whereas for an uninfected cell, we have *P*(*Z*_*k*_ = 0) = *p*_0_ and *P*(*Z*_*k*_ = 1) = *p*_1_. Here, *p*_0_ is the probability that the signaling pathway has not been activated by the pathogen and *p*_0_ + *p*_1_ = 1. This is exactly the above situation of a hidden combinatorial knock-down and the additional model parameter *p*_*j*_, *j* ∊ {0, 1} either needs to be estimated for each observation, or it has to be integrated out.

#### Learning from single-cell data

As illustrated in the infection experiment example above, in general, the signaling state *Z*_*k*_ can be different for each individual cell *c* in an experiment *k*, and therefore we have to treat each cell as an individual observation. Regarding single cells as independent, for a given network structure Φ, the local likelihoods further decompose into

$$\begin{array}{c} P\left(d_{ek}|\Phi ,\theta_{e}=s\right)=\prod_{c=1}^{c_{k}}P\left(d_{ekc}|\Phi ,\theta_{e}=s\right), \end{array}$$ (18)

where *c*_*k*_ is the number of cells in experiment *k*. Instead of a single number, now *S*_*sk*_ = (*S*_*skc*_)_*c* = 1, …, *c*_*k*__ is a vector where each *S*_*skc*_ is the state of gene *s* in cell *c* under knock-down *k*. With this modification, the marginal likelihood expands to

$$\begin{array}{c} P\left(D|\Phi \right)=\prod_{e=1}^{m}\sum_{s=1}^{n}P\left(\theta_{e}=s\right)\prod_{k=1}^{K}\prod_{c=1}^{c_{k}}\sum_{j∊\{0,1\}}p_{j}P\left(d_{ekc}|\Phi ,\theta_{e}=s,Z_{kc}=j\right). \end{array}$$ (19)

In other words, for each cell, there will be an effect of the perturbation set **𝓢**_*k*_ on feature *e* if any of the perturbations reach gene *s* and *e* is connected to *s*.

#### Model identifiability

As shown in [36], NEMs have unidentifiable components. If two nodes share the same set of parents, then these two nodes are indistinguishable. Furthermore, NEMs are unique only up to reversals, i.e., different parametrizations can exist for the same model that explain the data equally well. Such equivalent representations are related by cyclic node permutations, ΦΘ = Φ′Θ′ with (Φ′, Θ′) = (Φ*S* ^−1^, *S*Θ) and permutation matrix *S* reversing cycles in Φ. This result still holds when adding the additional hidden pathway state *Z*.

Regarding inference of the new parameter *p*_0_, there are only few situations in which *p* cannot be learned. For a NEMix model with given graphs Φ and Θ, the pathway inactivation probability *p*_0_ of its hidden pathway activity *Z* is not identifiable, if and only if either (1) there are no observables from 𝓔 attached downstream of *Z*, or (2) *Z* is connected only to sub-components of a network that are always perturbed (for a proof, see ‘Unidentifiable parameters’ in S1 Text). Both conditions are rather artificial cases. The first one describes the situation where some signaling genes do not have any features attached and *Z* is connected only to these. In this case, there are no observations from which *p*_0_ can be estimated. However, usually we assume a uniform attachment of features to the genes and models containing genes without any downstream features are hardly ever observed. The interpretation of the second condition is that only genes which receive a propagated signal from all other genes in the network are affected by the pathway deactivation. Here, *p*_0_ cannot be estimated because all observations downstream of *Z* will show an effect, independent of the state of *Z*. Again, it appears rather exceptional that only the final node of a signaling cascade is affected by a pathway deactivation.

### NEMix inference

Structure learning is performed using a greedy heuristic to find an optimal network. Similar to the NEM procedure described in [3], edges are incrementally added if the likelihood is increased (see ‘Structure learning’ in S1 Text). In addition, our approach is restricted to structures without incoming edges into the hidden root *Z*. We initialize the algorithm with a set of initial networks. These consist of the empty graph and one edge connecting *Z* to one of the knock-down genes. Additionally, we limit the out-degree of *Z* to two. Here, by out-degree we mean only the non-transitive edges. We still allow the insertion of transitive edges from *Z* to any signaling gene, which has to be added in order to fulfill the transitivity requirement. This regularization reduces the search space and prevents that too many dependencies between genes are explained by *Z* alone.

As for classic NEMs, network structure scoring involves the marginal likelihood. For the NEMix model, *P*(**𝒟**∣Φ) cannot be optimized analytically. Marginalization over the feature attachments is omitted in our extended model. Instead, we estimate *θ* jointly with *p* during model inference. To do so, we approximate the marginal likelihood (10) by the expectation of the complete data log-likelihood

$$\begin{array}{c} P\left(D,Z|\Phi ,\theta ,p\right)=\prod_{k=1}^{K}\prod_{c=1}^{c_{k}}\prod_{j∊\{0,1\}}{\left[p_{j}\prod_{e=1}^{m}P\left(d_{ekc}|\Phi ,\theta_{e}=s,Z_{kc}=j\right)\right]}^{Z_{kc}^{\left(j\right)}}, \end{array}$$ (20)

with respect to *Z*, where *θ* and *p*_0_ need to be efficiently estimated. For this task we have developed an EM algorithm. A derivation of the expected hidden log-likelihood and the maximum likelihood estimates is given in ‘Estimating the hidden signal’ of S1 Text. When starting the EM algorithm, *p*_0_ is initialized with a random draw from the uniform distribution and for *θ* we use a uniform initial configuration.

### Implementation

The NEMix model is included as part of the R/Bioconductor package NEM as an additional inference type. It is invoked by calling the package’s main function NEM(data, inference = ‘NEM.greedy’, control) and choosing the inference type control$type = ‘NEMix’. (See ‘NEMix implementation in NEM package’ in S1 Text for more detailed instructions on the implementation and usage of NEMix in R). To record run-times of NEMix model estimation, simulations were run without any parallelization on a 1.7GHz Intel i7 machine. Only one starting configuration was used, and EM iterations were performed using three restarts to avoid local optima that are globally suboptimal. For realistic data sets of 300 features and 200 cells per knock-down, NEMix estimation took on average nine minutes for 5-gene networks, with an average of 13 iteration steps until convergence of the EM algorithm. For the 8-gene network, the average run-time was 66 minutes, while the average number of iterations per EM round remained 13 also for these larger networks. The longer run-times of NEMix models as compared to NEMs are primarily due to the hidden data estimation. Each structure scored once in a NEM inference, needs to be scored 40 times on average during NEMix estimation. In addition, the input data sets are roughly 200 times larger.

## Supporting Information

S1 Text Supplementary texts. The supplementary text contains additional information regarding the NEMIX model, description and pre-processing of the data sets, as well as a short usage description of the NEMIX code in the R package nem.
(PDF)

S1 Fig Sample networks. All 30 sample networks were randomly generated from the KEGG graph using a random walk along the edges. Unidentifiable structures were omitted. The blue node marks the randomly added hidden signal.
(EPS)

S2 Fig Performance for varying *p*_0_. For each of the 30 generated networks, 50 data set were drawn. In (A) the area under the ROC curve (AUC) was calculated based on edge frequencies of the samples. The right most panel displays the result for all values of *p*_0_ jointly. Sub-figure (B) shows the area under the PR curve (AUPRC).
(EPS)

S3 Fig Accuracy values per network. For each of the 30 generated networks, 50 data sets were drawn. Then, the accuracy (ACC) was calculated based on edge frequencies of the samples.
(EPS)

S4 Fig Estimated values for *p*_0_. For each of the 30 generated networks, 50 data sets were drawn. The distribution of the estimated *p*_0_ per network is shown. Each row represents a different true signal disruption probability.
(EPS)

S5 Fig Inferred pathway states *Z*. For each of the 30 generated networks, 50 data sets were drawn. Percentage of correctly inferred state values of *Z* for the sample data sets is shown for each of generated networks.
(EPS)

S6 Fig Performance for larger network sizes. To assess the edge recovery performance for larger NEMix models, we ran a reduced simulation study. We sampled 30 random networks of network size *n* = 5, 10, and 15 genes. The hidden variable *Z* was again attached randomly to at most 2 of the signaling genes (plus additional transitive edges). We fixed *p*_0_ to 0.4, which is close to our application example. For each network, we then generated 30 data sets of 300 features from 200 cells per each knock-down. For run-time reasons we only initiated the structure search with the empty network and used just 2 restarts for the EM runs. This reduces performance of network learning but shows how the overall performance scales with growing network size. Even for larger networks performance is still very good as can be seen from the area under ROC curve (A), area under precision-recall curve (AUPRC; B) and accuracy (C). Estimation of *p*_0_ becomes even more precise for larger *n*, as shown in (D) by the absolute distance of the sampled from the estimated *p*_0_. Run-time on the other hand increases substantially for larger networks. Panel (E) shows the run-times per network estimation in minutes.
(EPS)

S7 Fig Performance of feature attachments *θ*. For each of the 30 generated networks, 50 data sets were drawn. In (A) the percentage of correctly inferred feature attachments are displayed and (B) shows the percentage of correctly filtered uninformative features. Both plots show results for different fractions of signal disruption *p*_0_.
(EPS)

S8 Fig Network wise performance of feature attachments *θ*. For each of the 30 generated networks, 50 data sets were drawn. In (A) the percentage of correctly inferred feature attachments are displayed and (B) shows the percentage of correctly filtered uninformative features, for each individual network.
(EPS)

S9 Fig GSEA for reliable siRNAs. To see if KEGG pathways are affected differently by off-targeting siRNAs, we performed a gene set enrichment analysis [32] on the siRNA scores, using the implementation in the R package ‘HTSanalyzR’ [37].
(EPS)

S10 Fig Inferred 8 gene MAPK networks on HRV infection data. Best networks of the 8 top scoring siRNAs from the MAPK pathway for HRV infection for the different compared methods are displayed. (A) shows the known KEGG pathway. (B) is the inferred NEM and (C) the sc-NEM. (D) left shows the known network with the most likely attachment of the hidden variable *Z* (blue) and (E) is the inferred NEMix. For all networks their performance is summarized in S1 Table.
(EPS)

S11 Fig Performance of MAPK network inference. We computed the specificity (A) and sensitivity (B) for all compared methods, based on 50 bootstrap samples. Both plots show the results for 5 and 8 signaling genes with top scoring siRNAs, using the HRV infection data. Sub-figure (C) shows robustness of inferred pathway activity. The estimated pathway activity for 5 and 8 gene networks, derived from the 50 bootstrap samples is shown. *p*_0_ shows little variation and is similar for both networks.
(EPS)

S12 Fig Consensus networks for MAPK pathway. Consensus networks for 5 genes (A) and 8 genes (B) are displays. Shown are all edges with frequency of at least 0.7 in the 50 bootstrap samples. NEMix inference was run using the 16 different starting configurations. For each bootstrap sample the best solution was chosen.
(EPS)

S13 Fig Feature attachments for the 5 MAPK pathway genes. Histograms show the selection frequencies for image features from 50 bootstrap samples on the HRV infection data.
(EPS)

S14 Fig Shared feature usage for compared methods. The Venn diagrams compare frequently attached features (left) and never attached features (right) based on the 50 bootstrap samples. Results for the 5 gene network are shown in the top row and for the 8 gene network in the bottom row.
(EPS)

S15 Fig Uninformative features for the 5-gene network. Each row shows the probability for one feature of being attached to each of the genes in the network or the null node. For all features in this plot, the null node had the highest probability, which means, they are filtered out. Features are colored by the channel they were measured from. These channels are, fluorescence of DNA in the nucleus (blue), fluorescence of actin (red), fluorescence of cell internal pathogens (green). Furthermore there are general location and orientation features (black). The measurements themselves then give information on intensity, shape, texture or neighbors of the objects segmented from the images. These objects are ‘Cells’: the cell body, ‘Nuclei’: the cell nuclei, ‘PeriNuclei’: a peripheral area around the nucleus, ‘VoronoiCells’: the area of the cell from a Voronoi-tessellation of the image. Many of the uninformative features are related to orientation of objects or their location, which are expected not to carry useful information for the network inference.
(EPS)

S16 Fig Feature attachments in the 5-gene network. Each row shows the probability for one feature of being attached to each of the genes in the network or the null node. Rows are sorted by the gene for which the attachment probability is highest. For a description of the different feature types see caption of supplementary S15 Fig.
(EPS)

S17 Fig Illustration of a NEMix, with unidentifiable *p* = *P*(*Z*). The hidden variable is attached only to signaling genes that are always perturbed. For models with such structure *p* cannot be inferred.
(EPS)

S1 Table Performance summary of the 8 gene MAPK network. The first column gives the log-likelihood for each model, showing that the true network is much less likely than the inferred networks. The second and third column show performance of the networks in terms of accuracy (ACC) and area under curve (AUC). The inferred *p*_0_ for the NEMix models is displayed in column four. Column five indicates the corresponding sub-figure of Fig. 3. The network ‘KEGG Graph + Z’ denotes the structure of the known KEGG network, where only the position of *Z, p*_0_, and *θ* are inferred.
(PDF)
