# Supplementary material for: NEMix: Single-cell Nested Effects Models for Probabilistic Pathway Stimulation
Source: PLoS Comput Biol. 2015 Apr 16;11(4):e1004078. doi: 10.1371/journal.pcbi.1004078 (PMC4400057; doi:10.1371/journal.pcbi.1004078)
Supplement: S1 Text — The supplementary text contains additional information regarding the NEMIX model, description and pre-processing of the data sets, as well as a short usage description of the NEMIX code in the R package nem. (PDF) [file pcbi.1004078.s001.pdf]

NEMix: Single-cell Nested Effects Models for Probabilistic Pathway Stimulation

Juliane Siebourg-Polster[1,2], Daria Mudrak[3], Mario Emmenlauer[4], Pauli Rämö[4], Christoph Dehio[4], Urs Greber[3], Holger Fröhlich[5], Niko Beerenwinkel[1,2,*]
1 Department of Biosystems Science and Engineering, ETH Zurich, Basel, Switzerland
2 SIB Swiss Institute of Bioinformatics, Basel, Switzerland
3 Institute of Molecular Life Sciences, University of Zurich, Zurich, Switzerland
4 Biozentrum, University of Basel, Basel, Switzerland
5 Algorithmic Bioinformatics, Bonn-Aachen International Center for IT, University of Bonn, Bonn, Germany
*** E-mail: niko.beerenwinkel@bsse.ethz.ch**

Supporting Information

Estimating the hidden signal

For a given structure Φ and fixed parameters θ, we obtain the likelihood of the observed data by marginalizing over the hidden variable Z,

$$\begin{aligned} P(D \mid \Phi, \theta) &= \sum_Z P(D, Z \mid \Phi, \theta) = \sum_{\substack{Z_1 \in \\ \{0,1\}^{c_1}}} \cdots \sum_{\substack{Z_K \in \\ \{0,1\}^{c_K}}} P(Z_k) P(D \mid Z_k, \Phi, \theta) \\ &= \sum_{\substack{Z_1 \in \\ \{0,1\}^{c_1}}} \cdots \sum_{\substack{Z_K \in \\ \{0,1\}^{c_K}}} \prod_{k=1}^{K} \prod_{c=1}^{c_k} P(Z_{kc}) \prod_{e=1}^{m} P(d_{ekc} \mid \Phi, \theta_e = s, Z_{kc} = j). \end{aligned} \tag{1}$$

Apart from the now fixed feature connections θ, this is equivalent to the likelihood (6), where all the summations over the possible states of Z are taken out of the products over all the observations. Maximizing this equation is computationally difficult and we therefore develop an EM algorithm [3] using the complete data likelihood

$$P(D, Z \mid \Phi, \theta) = \prod_{k=1}^{K} \prod_{c=1}^{c_k} P(Z_{kc}) \prod_{e=1}^{m} P(d_{ek} \mid \Phi, \theta_e = s, Z_{kc} = j). \tag{2}$$

Let $Z_{kc}^{(j)}$ be the indicator variable that Z_{kc} is in state j for experiment k in cell c.

$$Z_{kc}^{(j)} = \begin{cases} 1 & Z_{kc} = j \\ 0 & otherwise. \end{cases} \tag{3}$$

Then we can rewrite (2) as

$$P(D, Z \mid \Phi, \theta, p) = \prod_{k=1}^{K} \prod_{c=1}^{c_k} \prod_{j \in \{0,1\}} \left[p_j \prod_{e=1}^{m} P(d_{ekc} \mid \Phi, \theta_e = s, Z_{kc} = j) \right]^{Z_{kc}^{(j)}}. \tag{4}$$

As introduced in the first section, the connections of observed features to the genes (θ) are often integrated out. However, apart from being a time consuming step, the introduction of the sum over all signaling genes into the complete data likelihood (2) makes this function hard to optimize

$$P(D, Z \mid \Phi) = \prod_{e=1}^{m} \sum_{s=1}^{n} P(\theta_e = s) \prod_{k=1}^{K} \prod_{c=1}^{c_k} \prod_{j \in \{0,1\}} \left[(p_j)^{\frac{1}{m}} P(d_{ekc} \mid \Phi, \theta_e = s, Z_{kc} = j) \right]^{Z_{kc}^{(j)}}. \tag{5}$$

Another possibility is to learn the probability of each edge $\theta_e = s$ jointly with p using an EM algorithm. In the E step, we calculate the expected complete data log likelihood w.r.t. the posterior of the hidden variable

$$P(Z \mid D, \Phi, \theta^{old}, p^{old}) = \frac{P(D, Z \mid \Phi, \theta^{old}, p^{old})}{\sum_Z P(D, Z \mid \Phi, \theta^{old}, p^{old})}, \tag{6}$$

for the current parameter setting denoted with p^{old}, θ^{old}. We obtain

$$\begin{aligned} \mathcal{Q}(p, \theta; p^{old}, \theta^{old}) &= \mathbb{E}_{P(Z|D,\Phi,\theta^{old},p^{old})}[\log P(D, Z \mid \theta, \Phi, p)] \\ &= \mathbb{E}\left[\sum_{k=1}^{K} \sum_{c=1}^{c_k} \sum_{j \in \{0,1\}} Z_{kc}^{(j)} \left(\log(p_j) + \sum_{e=1}^{m} \log\left(P(d_{ekc} \mid \Phi, \theta_e = s, Z_{kc} = j) \right) \right) \right] \\ &= \sum_{k=1}^{K} \sum_{c=1}^{c_k} \sum_{j \in \{0,1\}} \gamma_{kc}^{(j)} \log(p_j) + \gamma_{kc}^{(j)} \sum_{e=1}^{m} \log\left(P(d_{ekc} \mid \Phi, \theta_e = s, Z_{kc} = j) \right), \end{aligned} \tag{7}$$

where for each cell c in experiment k $\gamma_{kc}^{(j)}$ is the responsibility of pathway (in-)activation for observation d_{ekc},

$$\begin{aligned} \gamma_{kc}^{(j)} &= \mathbb{E}[Z_{kc}^{(j)} = 1 \mid d_{kc}, \Phi, \theta, p] \\ &= \frac{p_j \cdot P(d_{kc} \mid \Phi, \theta)}{\sum_{j'} p_{j'} \cdot P(d_{kc} \mid \Phi, \theta)} \\ &= \frac{p_j \prod_{e=1}^{m} P(d_{ekc} \mid \Phi, \theta_e = s, Z_{kc} = j)}{\sum_{j'} p_{j'} \prod_{e=1}^{m} P(d_{ekc} \mid \Phi, \theta_e = s, Z_{kc} = j')}. \end{aligned} \tag{8}$$

In fact, this expected value has to be calculated only for cells that were not infected. For infected cells, $Z_{kc} = 1$ is observed and the E step is not needed.

In the M step $\mathcal{Q}(p, \theta; p^{old}, \theta^{old})$ is first maximized w.r.t. p_j. This leads to

$$\hat{p}_j = \frac{1}{N} \sum_{k=1}^{K} \sum_{c=1}^{c_k} \gamma_{kc}^{(j)}, \tag{9}$$

where $N = \sum_{k=1}^{K} \sum_{c=1}^{c_k} 1$ is the total number of observations (summing the cells of all experiments).

Since the θ_e are independent and discrete, maximization of $\mathcal{Q}$ w.r.t. θ is achieved by iteratively selecting the signaling gene s that maximizes

$$\hat{\theta}_e = \underset{s \in \{1,\ldots,n\}}{\operatorname{argmax}} \sum_{k=1}^{K} \sum_{c=1}^{c_k} \sum_{j \in \{0,1\}} \gamma_{kc}^{(j)} \log\left(P(d_{ekc} \mid \Phi, \theta_e = s, Z_{kc} = j) \right). \tag{10}$$

Maximum likelihood estimates for p_j and θ

Given the expected hidden log-likelihood

$$\mathcal{Q}(p,\theta;p^{old},\theta^{old}) = \sum_{k=1}^{K}\sum_{c=1}^{c_k}\sum_{j\in\{0,1\}} \gamma_{kc}^{(j)}\log(p_j) + \gamma_{kc}^{(j)}\sum_{e=1}^{m}\log\left(P(d_{ekc}\mid \Phi,\theta,Z_{kc}=j)\right), \tag{11}$$

in the M step, $\mathcal{Q}(p,\theta;p^{old},\theta^{old})$ is maximized w.r.t. each p_j. This involves only the left term in (11) and the constraint $p_0+p_1=1$ needs to be obeyed

$$\begin{aligned}\frac{d\mathcal{Q}(p,\theta;p^{old},\theta^{old})}{dp_j} &= \frac{d}{dp_j}\left(\sum_{k=1}^{K}\sum_{c=1}^{c_k}\gamma_{kc}^{(j)}\log(p_j)+\lambda((p_0+p_1)-1)\right)\\ &= \sum_{k=1}^{K}\sum_{c=1}^{c_k}\frac{\gamma_{kc}^{(j)}}{p_j}+\lambda = 0. \end{aligned}\tag{12}$$

Solving for p_j leads to

$$p_j = -\sum_{k=1}^{K}\sum_{c=1}^{c_k}\frac{\gamma_{kc}^{(j)}}{\lambda} \tag{13}$$

multiplying by λ and taking the sum over j on both sides results in

$$\lambda = -\sum_{k=1}^{K}\sum_{c=1}^{c_k}\sum_{j\in\{0,1\}}\gamma_{kc}^{(j)} = -\sum_{k=1}^{K}\sum_{c=1}^{c_k}1, \tag{14}$$

for the Lagrange multiplier λ. Defining $N=\sum_{k=1}^{K}\sum_{c=1}^{c_k}1$ as the total number of observations (summing the cells of all experiments), the final solution for p_j then is

$$p_j = \frac{1}{N}\sum_{k=1}^{K}\sum_{c=1}^{c_k}\gamma_{kc}^{(j)}. \tag{15}$$

Since the θ_e are independent and discrete, to maximize $\mathcal{Q}$ w.r.t. θ, we need to solve

$$\begin{aligned}&\underset{\theta_1,\ldots,\theta_m\in n^m}{\operatorname{argmax}}\ \mathcal{Q}(p,\theta;p^{old},\theta^{old})\\ &= \underset{\theta_1,\ldots,\theta_m\in n^m}{\operatorname{argmax}}\sum_{k=1}^{K}\sum_{c=1}^{c_k}\sum_{j\in\{0,1\}}\gamma_{kc}^{(j)}\sum_{e=1}^{m}\log\left(P(d_{ekc}\mid\Phi,\theta_e=s,Z_{kc}=j)\right)\\ &= \sum_{e=1}^{m}\underset{\theta_e}{\operatorname{argmax}}\sum_{k=1}^{K}\sum_{c=1}^{c_k}\sum_{j\in\{0,1\}}\gamma_{kc}^{(j)}\log\left(P(d_{ekc}\mid\Phi,\theta_e=s,Z_{kc}=j)\right).\end{aligned}\tag{16}$$

This equation is maximized by iteratively finding for each feature the gene that maximizes the above term.

Unidentifiable parameters

Proposition 1. *For a given NEMix model, the pathway activation probability p of its hidden pathway activity Z is not identifiable, if and only if either of the following holds*

1. *There are no observables from $\mathcal{E}$ attached downstream of Z.*

2. *Z is connected only to sub-components of a network that are always perturbed.*

Proof. It needs to be shown that the likelihood of a model is independent of p,if and only if one of the two cases is true.

Let $\mathcal{S}$ be the set of genes and partition it into subsets $\mathcal{S}_d$ which are always perturbed and the rest $\mathcal{S}_u$. Then, $\mathcal{S}_d$ contains nodes downstream of all other nodes in $\mathcal{S}$, and if $|\mathcal{S}_d| > 1$ then $\mathcal{S}_d$ is a fully connected component. Let furthermore $\mathcal{K}$ be the set of perturbation experiments and $\mathcal{E} = \{\mathcal{E}_u, \mathcal{E}_d\}$ the observed features attached to the according group of genes.

$\Rightarrow$: Assuming the case that Z is only connected to $\mathcal{S}_d$ for a given NEMix with signal graph Φ and feature connections θ, the likelihood function

$$P(D \mid \Phi, \theta) = \prod_{\mathcal{E}} \prod_{\mathcal{K}} \sum_{j \in \{0,1\}} p_j \cdot P(d_{ek} \mid \Phi, \theta, Z_k = j) \tag{17}$$

can be partitioned in the following way:

$$\begin{aligned} P(D \mid \Phi, \theta) &= \prod_{\mathcal{E}_u} \prod_{\mathcal{K}} \sum_{j \in \{0,1\}} p_j \cdot P(d_{ek} \mid \Phi, \theta, Z_k = j) \cdot \prod_{\mathcal{E}_d} \prod_{\mathcal{K}} \sum_{j \in \{0,1\}} p_j \cdot P(d_{ek} \mid \Phi, \theta, Z_k = j) \\ &= \prod_{\mathcal{E}_u} \prod_{\mathcal{K}} \sum_{j \in \{0,1\}} p_j \cdot P(d_{ek} \mid \Phi, \theta) \cdot \prod_{\mathcal{E}_d} \prod_{\mathcal{K}} \sum_{j \in \{0,1\}} p_j \cdot \sum_{S_{sk} \in \{0,1\}} P(d_{ek} \mid \theta, S_{sk}) P(S_{sk}) \end{aligned} \tag{18}$$

In the first part of the product, the local effect probabilities for all $e \in \mathcal{E}_u$ are independent of Z since there are no edges connecting Z to features in $\mathcal{E}_u$. In the second part of the product, we expand the local effect likelihoods by the marginal probability of the states of the node, which the feature is attached to (as introduced in equation 7 in the paper). Since $S_{sk} \in \mathcal{S}_d$, we have $P(S_{sk} = 0) = 1$ for all $k \in \mathcal{K}$. Together with the constraint $p0 + p1 = 1$, it follows

$$\begin{aligned} P(D \mid \Phi, \theta) &= \prod_{\mathcal{E}_u} \prod_{\mathcal{K}} P(d_{ek} \mid \Phi, \theta) \left(\sum_{j \in \{0,1\}} p_j \right) \cdot \prod_{\mathcal{E}_d} \prod_{\mathcal{K}} P(d_{ek} \mid \theta, S_{sk} = 0) \left(\sum_{j \in \{0,1\}} p_j \right) \\ &= \prod_{\mathcal{E}_u} \prod_{\mathcal{K}} P(d_{ek} \mid \Phi, \theta) \cdot \prod_{\mathcal{E}_d} \prod_{\mathcal{K}} P(d_{ek} \mid \theta, S_{sk} = 0), \end{aligned} \tag{19}$$

which is independent of p. Given the case that there are no observables downstream of Z, all $e \in \mathcal{E}_u$ and the above equation reduces to only the first product.

$\Leftarrow$: Assuming there is a connection from Z to $e_i \in \mathcal{E}_u$, we expand equation 19 using the state variables

$$\begin{aligned} P(D \mid \Phi, \theta) = &\prod_{\mathcal{E}_u \setminus e_i} \prod_{\mathcal{K}} P(d_{ek} \mid \Phi, \theta) \cdot \prod_{\mathcal{K}} p_j \cdot \sum_{S_{sk} \in \{0,1\}} P(d_{e_i k} \mid \theta_{e_i s}, S_{sk}) P(S_{sk} \mid Z_k = j) \cdot \\ &\prod_{\mathcal{E}_d} \prod_{\mathcal{K}} P(d_{ek} \mid \theta_e = s, S_{sk} = 0). \end{aligned} \tag{20}$$

For the gene S_s, connecting Z to the feature e_i, there must be at least one experiment k for which $P(S_{sk} = 0) \neq 1$, because otherwise $S_s \in \mathcal{S}_d$. Since $Z \in pa(S_s)$, $P(d_{e_i k} \mid \theta_{e_i s}, S_{sk})$ is depending on Z. p would only be unidentifiable if $P(d_{e_i k} \mid \theta_{e_i s}, S_{sk} = 1)$ is equal to $P(d_{e_i k} \mid \theta_{e_i s}, S_{sk} = 0)$, which is generally not the case. □

Even if p_0 is identifiable, there exist some cases where $p_0 = P(Z = 0)$ can only poorly be estimated. When Z is connected to genes that have only few features attached, and the true p_0 is high, there is not enough evidence in the data to recover the true states of Z. Also, if most features are downstream of almost always silenced genes, states of Z are badly recovered.

Modeling the effect likelihoods

The set of local effect likelihoods used for scoring the NEMix structures, consists of a set of matrices, one per knock-down k. Each matrix has one column per per observed cell with one entry for each measured feature, denoted with $\mathcal{D}_k$. These local effect likelihoods can be pre-computed from the data.

To estimate the local effect likelihoods we used log odds scores [7]. As mentioned in the simulation study, no controls are available for the experimental data. For each measured image feature, we obtained a negative control distribution, F_0, by sampling from all cells of the plate containing the knock-down well, under the assumption that the majority of cells over a plate will not show any effects. For the effect distribution, F_E, of a feature, the values of all cells within a knock-down experiment were taken. The control and effect distributions were estimated with the 'kernel cumulative function estimate' from the R CRAN package 'ks' [8]. Then, for each cell c in experiment k and each feature e, we calculate the log odds scores

$$R_{ekc} = \log \frac{P(d_{ekc} \mid F_{E_k})}{P(d_{ekc} \mid F_0)}. \tag{21}$$

If a knock-down has a strong effect on a feature, then R_{ekc} will be much larger than zero. If there is no overall effect, control and effect distribution will be very similar and $R_{ekc} \approx 0$.

Structure learning

Structure inference to find an optimal network for transitively closed networks is performed using a greedy heuristic as described in [1]. In this approach, edges are incrementally added until the likelihood score cannot be increased anymore.

An algorithm already implemented in the R package `nem` is used for NEMix inference, with slight adjustments. The procedure is initiated with a given network structure. Then, all networks with one additional edge are built and their transitive closure is computed. Finally, the structure with the highest likelihood score is selected and the procedure is repeated. The algorithm terminates when no edge insertion can increase the likelihood anymore. Edge deletion and reversal steps are not performed since these may result in structures that are not transitively closed.

In addition to the existing procedure, our approach is restricted to structures without incoming edges into the hidden root vertex Z. We furthermore initialize the algorithm with a set of initial networks. Each of these networks consists of the empty signal graph and one of all possible combinations of connecting Z to the knock-down genes. This approach is only feasible for a small number of nodes, because it results in 2^n different starting networks. For larger networks, we only initialize with Z attached to each of the genes individually. Additionally, the out-degree of Z can be limited, where out-degree refers here only to the non-transitive edges from Z to any of the other nodes. In our examples, we limited the out-degree of Z to two edges. This regularization reduces the search space and prevents that too many dependencies between genes are explained by Z alone.

Dataset preparation and description

Data of the HRV infection example stem from a genome wide RNAi HRV infection screen (unpublished data). We extracted the data for the 8 genes used in this study. It is available in the supplementary data set. Genes are named by their Entrez ID. The genome wide RNAi screen followed the protocol in [2], which is summarized in the following. HeLa cells were infected with HRV to study their entry mechanism and to

identify host cell genes involved in HRV infection. The screen was performed using a genome wide pooled siRNA library (Dharmacon Smart Pool). On 384-well plates, approximately 1000 cells per well were transfected with siRNAs and after transfection cells were infected with HRV for 7 hours. Afterwards, an assay of automated microscopy and automated image analysis was used, taking nine images per well. In the image analysis cells were identified by the cell nuclei (measured by DAPI staining of the DNA). Viruses were identified using intensity of GFP expressed by the viruses, and the Actin skeleton of a cell was stained with Cy5. Images were analyzed using the software CellProfiler [5]. The resulting data sets consisted of 360 cell features from 9 images per knock-down experiment. They are arranged in three rows and three columns. To avoid too many out-of-focus cells, we used only the middle image, as it showed to have highest quality. In this way we avoided too many out-of-focus cells. After filtering we each knock-down had values between 200 to 300. The screen lacked reliable controls and therefore for each gene, we generated a negative control distribution as a random sample of cells, distributed over the whole plate. This is done under the assumption that the majority of cells on a plate will not show an effect in a certain feature. For the NEM model knock-down populations were compared feature wise to the control distributions, using a Wilcoxon test. From the resulting p-values effect strength was estimated using a Beta-Uniform-Mixture model [4]. For the NEMix and sc-NEM model we used log likelihood ratios for the individual cells as described in the main text.

siRNA filtering for off-targets

siRNAs are known to be very prone to off-target effects as they tend to mimic microRNAs and reproduce their effects [6]. To reduce this confounding source as much as possible, we ranked the genes according to their siRNAs amount of off-target effects. The ranking was conducted using the microRNA target prediction matrix from the tool 'Target Scan' [9]. For each siRNA, it provides weights for every gene, indicating how much the siRNA contributes to the knock-down of the gene. The weights are calculated as the percentage of transcript repression that the siRNA induces for a gene. Since the screens were performed with four pooled siRNAs, we averaged over the individual siRNA vectors. From the resulting vectors of weights we calculated an L^2 siRNA score for each gene as

$$score(\text{siRNA}) = \sum_{i=1}^{n} w_i^2, \qquad (22)$$

where n is the total number of genes that can be targeted and w_i the transcript repression percentage for gene i. In this way, strong off-targets are penalized by receiving a high score.

NEMix implementation in NEM package

The `NEMix` procedure is implemented in the existing R Bioconductor package `NEM`. The main function to produce a NEM takes a data matrix, a set of options and an inference type. `NEMix` is invoked by setting the inference type `type='NEMix'`. Additionally, several other options can be given. First, the input dataset `D` must be loaded. It needs to be a matrix with the observables as rows and each experiment (i.e., each 'cell') is a column. The 5 gene HRV infection dataset is already contained in the package.

```
library(NEM)
data(HRV_MAPK_RNAi)
dim(HRV_MAPK_RNAi.dens)
dim(HRV_MAPK_RNAi.llr)
```

`HRV_MAPK_RNAi.dens` contains the log p-value densities for classic NEM estimation. `HRV_MAPK_RNAi.llr` contains the single cell log odds ratios used for the NEMix inference. In the following the NEMix inference is lined out on the HRV data.

```
D<-HRV_MAPK_RNAi.llr
```

Now the experiment names and gene names are set. In the following example, column names are the names of the knock-down genes followed by the index of the cell (e.g. `geneA.1`).

```
experiments <- colnames(D)
knockdowns  <- sapply(colnames(D),function(x){strsplit(x,'\\.')[[1]][1]})
Sgenes      <- unique(knockdowns)
n <- length(Sgenes)
```

The next line initializes a list with all default parameters for the model.

```
control <- set.default.parameters(Sgenes)
```

To run the 'NEMix' version, the correct inference type and a name for the hidden signal needs to be set.

```
control$type         <- 'NEMix'
control$hiddenSignal <- 'Z'
```

Since there is more than one observation per knock-down, and there could be several knock-downs per cell, we need to map knock-downs to experiments.

```
control$map  <- as.list(knockdowns)
```

Additionally, the following parameters, concerning EM-restarts, a prior for p_0, known states for Z and maximal out-degree for Z can be given.

```
control$NEMix.restarts     <- 3
control$p0                 <- 0.5
control$knownZ             <- NA
control$maxOutDegreeZ      <- 2
control$maxStartOutDegreeZ <- 2
```

Finally, the NEM function can be run. For structure inference there are two options. Either the exhaustive search (`inference='search'`) can be run for networks up to 4 nodes, or a greedy hill climber (`inference='NEM.greedy'`) can be applied.

```
NEMix <- nem(D=D, inference='NEM.greedy',control=control,verbose=FALSE)
```

The resulting list contains the results for the used initial attachments of Z. To get the optimal network on needs to look at the likelihood.

```
winner <- which.max(sapply(NEMix,function(x){x$llr}))
```

Finally we can plot the winning network

```
plot(NEMix[[winner]]$graph,main='MAPK nemix')
```

Hidden-root networks can be generated exhaustively up to 4 nodes using

```
models <- enumerate.hidden.models(Sgenes)
```

For simulation purposes, we additionally implemented a function to attach a random hidden signal to a network with a desired out-degree.

```
Phi <- sampleRndNetwork(1:5)
PhiZ <- generateHiddenSignal(Phi, maxOutDegreeZ=2)
```

Furthermore, there is a function for data simulation from a given NEMix structure.

```
sdata <- sampleNEMixData(PhiZ, hiddenSignal='Z', m=1:100, p0=0.3,
                  uninformative=10, type='lodds')
D <- sdata$D
```

On the implementation level, the main NEM-package workflow is adjusted by additional checks to handle the learning type `'NEMix'` correctly. All functions necessary for NEMix-inference can be found in the file `NEMix.R`.

References

1. Fröhlich H, Tresch A, Beissbarth T (2009) Nested effects models for learning signaling networks from perturbation data. Biometrical J 51: 304–323.
2. Rämö P, Drewek A, Arrieumerlou C, Beerenwinkel N, Ben-Tekaya H, et al. (2014) Simultaneous analysis of large-scale RNAi screens for pathogen entry. BMC Genomics accepted.
3. Dempster AP, Laird NM, Rubin DB (1977) Maximum likelihood from incomplete data via the EM algorithm. J R Stat Soc Ser B (Statistical Methodol 39: 1–38.
4. Fröhlich H, Fellmann M, Sueltmann H, Poustka A, Beissbarth T (2007) Large scale statistical inference of signaling pathways from RNAi and microarray data. BMC Bioinformatics 8: 386.
5. Carpenter AE, Jones TR, Lamprecht MR, Clarke C, Kang IH, et al. (2006) CellProfiler: image analysis software for identifying and quantifying cell phenotypes. Genome Biol 7: R100.
6. Seinen E, Burgerhof JGM, Jansen RC, Sibon OCM (2010) RNAi experiments in D. melanogaster: solutions to the overlooked problem of off-targets shared by independent dsRNAs. PLoS One 5: 1–7.
7. Tresch A, Markowetz F (2008) Structure learning in nested effects models. Stat Appl Genet Mol Biol 7: 26.
8. Duong T (2013). ks: Kernel smoothing. R package. URL `http://cran.r-project.org/package=ks`.
9. Lewis BP, Shih Ih, Jones-Rhoades MW, Bartel DP, Burge CB (2003) Prediction of Mammalian MicroRNA Targets. Cell 115: 787–798.
