# Supplementary material for: NEMix: Single-cell Nested Effects Models for Probabilistic Pathway Stimulation
Source: PLoS Comput Biol. 2015 Apr 16;11(4):e1004078. doi: 10.1371/journal.pcbi.1004078 (PMC4400057; doi:10.1371/journal.pcbi.1004078)
Supplement: S1 Table — The first column gives the log-likelihood for each model, showing that the true network is much less likely than the inferred networks. The second and third column show performance of the networks in terms of accuracy (ACC) and area under curve (AUC). The inferred p 0 for the NEMix models is displayed in column four. Column five indicates the corresponding sub-figure of Fig. 3. The network ‘KEGG Graph + Z’ denotes the structure of the known KEGG network, where only the position of Z, p 0, and θ are inferred. (PDF) [file pcbi.1004078.s019.pdf]

Supplementary Tables

Table S1. Performance summary of the 8 gene MAPK network. The first column gives the log-likelihood for each model, showing that the true network is much less likely than the inferred networks. The second and third column show performance of the networks in terms of accuracy (ACC) and area under curve (AUC). The inferred p_0 for the NEMix models is displayed in column four. Column five indicates the corresponding sub-figure of figure 3. The network 'KEGG Graph + Z' denotes the structure of the known KEGG network, where only the position of Z, p_0, and θ are inferred.

Network	Likelihood	ACC	AUC	p0	Sub-figure
True Graph	3717.26	1	1		A
NEM	6218.67	0.45	0.45		B
sc-NEM	55502.79	0.66	0.45		C
True Graph + Z	65447.15	1	1	0.52	D
NEMix	69189.39	0.86	0.8	0.54	E
